# Role of midline raphe in compartmental surgery for squamous cell carcinoma of the tongue

**DOI:** 10.1007/s00405-024-08929-x

**Published:** 2024-09-04

**Authors:** Grammatica Alberto, Ravanelli Marco, Tomasoni Michele, Conti Carlo, Pinacoli Aurora, Bozzola Anna, Farina Davide, Mattavelli Davide, Piazza Cesare

**Affiliations:** 1 Unit of Otorhinolaryngology – Head and Neck Surgery, ASST Spedali Civili di Brescia, Piazza Spedali Civili 1, 25123 Brescia, Italy; 2 Unit of Radiology, ASST Spedali Civili di Brescia, Brescia, Italy; 3 Department of Medical and Surgical Specialties, Radiological Sciences, and Public Health, School of Medicine, University of Brescia, Brescia, Italy; 4 Unit of Pathology, ASST Spedali Civili di Brescia, Brescia, Italy

**Keywords:** Oral cancer, Compartmental surgery, Tongue cancer, Midline raphe, Depth of infiltration

## Abstract

**Purpose:**

In the present study, we investigated how tumor distance from midline (TDFM) and depth of invasion (DOI) may affect survival outcomes after compartmental tongue surgery (CTS) for oral tongue squamous cell carcinoma (OTSCC).

**Methods:**

A retrospective series of cT2-T3 OTSCC treated with upfront CTS at our Department from 2010 to 2021 was evaluated. Radiological and pathological DOI and TDFM were correlated. The main outcomes were overall (OS) and loco-regional recurrence free survival (LRRFS). The linear relationship between DOI and TDFM with 2-year OS and LRRFS was tested. Survival estimates were obtained by the Kaplan-Meier method. Univariate analysis was performed for variables of interest, and results expressed in terms of hazard ratios and 95% confidence intervals.

**Results:**

A total of 64 patients underwent CTS and neck dissection. No significant difference was found between pathological (pDOI) and radiological DOI (rDOI) (*p* = 0.321) or between pathological (pTDFM) and radiological TDFM (*p* = 0.435). Two- and 5-year OS and LRRFS were 85.7% and 70.4%, 84.3% and 76.1%, respectively. A linear and significant relationship with OS (*p* = 0.020) and LRRFS (*p* = 0.013) was found for pDOI; although linear, the relationship between pTDFM was not statistically significant for either survival outcomes. Once categorized, the ideal cut-off for pDOI according to OS was set at 10 mm (*p* = 0.023).

**Conclusion:**

In patients undergoing CTS for primary OTSCC, magnetic resonance-derived rDOI represents an accurate estimate of pDOI, In contrast, TDFM was not associated with OS suggesting that the median raphe is a safe deep margin for CTS.

**protocol n.:**

BS/231,009 retrospectively registered.

## Introduction

The oral tongue is a muscular structure anatomically divided into two symmetric compartments by the midline lingual raphe. Whenever this structure is macroscopically involved and/or transgressed, an increased risk of contralateral neck metastasis is described, with a consequent detrimental effect on survival [1–3]. According to these premises, it is well accepted that depth of infiltration (DOI) is one of the most important negative prognosticators for loco-regional control and survival. Thus, according to the 8th Edition of the TNM staging system (8TNM) [4], this feature has been introduced to discriminate early (cT1-T2 for DOI < 10 mm) from more advanced cancers (cT3 for DOI ≥ 10 mm).

In the last decade, the concept of compartmental tongue surgery (CTS) for treatment of intermediate-advanced OTSCC has been proposed and popularized [5–7]. It is based on the evidence that: (1) a single hemilingual compartment is functionally independent from the opposite one; (2) extrinsic tongue muscles and intermingled vessels and nerves can act as pathways for local tumor spread; (3) the lymphatic connections between the oral tongue-floor of mouth and ipsilateral lymph nodes through the areolar tissue of the sublingual and submandibular areas constitute the T-N tract. The aim of this surgical technique is to remove the affected hemitongue compartment “en bloc” with the T-N tract and ipsilateral lymph nodes, following well-defined and easily reproducible anatomical landmarks [6]. The main criticism of CTS is its safety in considering the midline raphe as an oncologic barrier in terms of tumor spread to the contralateral compartment, and thus a safe margin for medial resection regardless its distance from the tumor, especially when this approaches the midline.

To tackle this issue, two questions should be addressed: (1) Does the distance between tumor margin and median raphe have a prognostic significance in CTS for OTSCC? (2) Does the median lingual raphe play a protective role against tumor progression towards the opposite compartment?

The aim of this study was therefore to evaluate a cohort of patients affected by cT2-T3 OTSCC and treated by upfront CTS, focusing on oncological outcomes in terms of overall (OS) and loco-regional recurrence free survival (LRRFS), analyzing how DOI and tumor distance from midline (TDFM) affect the outcomes of interest.

## Materials and methods

A retrospective analysis of consecutive patients affected by OTSCC treated by upfront surgery (with or without adjuvant treatments) from 2010 to 2021 was conducted at the Unit of Otorhinolaryngology – Head and Neck Surgery, ASST Spedali Civili of Brescia, University of Brescia, Italy.

Inclusion criteria were: (a) diagnosis of primary, treatment-naïve, cT2-T3 OTSCC; (b) upfront CTS with “en bloc” (at least unilateral) neck dissection with curative intent; (c) availability of preoperative magnetic resonance (MR) and histological slides of surgical specimens for revision. Exclusion criteria were: (a) tumor extensions to the contralateral tongue compartment, floor of mouth, oropharynx, hard palate, and mandible; (b) evidence of distant metastasis at diagnosis; (c) previous treatments for oral cancer.

Data on demographics, clinical, tumor-, and treatment-related characteristics were retrieved through consultation of medical records. Preoperative MR and histological specimens were reviewed by an experienced head and neck radiologist (M.R.) and pathologist (A.B.).

**Fig. 1:**
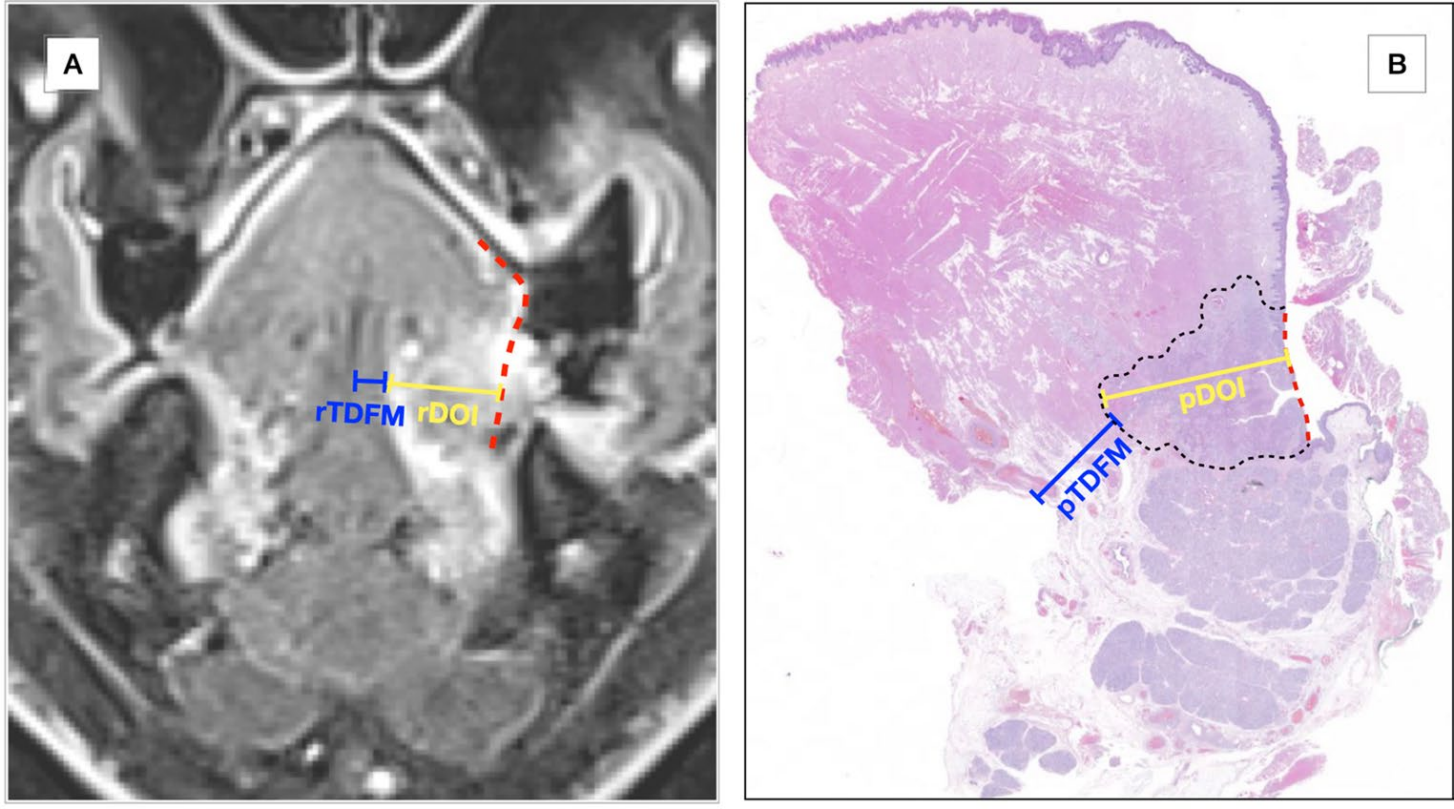
Graphical representation of the measurement of DOI and TDFM both pathologically and radiologically. Panel A: MR measurement of radiological depth of invasion (rDOI) (yellow) from the normal mucosal lining of the tongue (red dashed line) and radiological tumor distance from the midline (rTDFM) (blue). Panel B: measurement of pathological depth of invasion (pDOI) (yellow) from the normal mucosal lining of the tongue (red dashed line) and pathological tumor distance from the midline (pTDFM) (blue); tumor is contoured by black dashed line

DOI was defined as the distance between a tangential plane passing through the level of the normal basement membrane and the deepest point of tumor invasion [8]. On the other hand, TDFM was defined as the distance between the deepest point of tumor invasion and the midline lingual raphe (Fig. 1). Radiological depth of infiltration (rDOI) and radiological tumor distance from midline (rTDFM) were usually measured in the coronal plane on contrast-enhanced T1-weighted fat-saturated 3D sequences, thus allowing a measurement perpendicular to the tongue border.

The following data were revised:

- rDOI, measured on preoperative MR;
- pathological depth of infiltration (pDOI), measured on the histological slides of the surgical specimen;
- rTDFM, defined as the radiological distance from the deepest tumor margin to the lingual median raphe on preoperative MR;
- pathological tumor distance from midline (pTDFM), defined as the microscopic distance from the deepest tumor margin to the lingual median raphe;
- DOI difference (DOId), defined as the difference between rDOI and pDOI (rDOI-pDOI);
- TDFM difference (TDFMd), defined as the difference between rTDFM and pTDFM (rTDFM-pTDFM).

Local recurrences were recorded and defined as a SCC biopsy-proven tumor arising within 3 cm and > 3 years of time following the end of primary treatment [9].

This study was conducted in line with the principles of the Declaration of Helsinki. Ethical approval was obtained by the local ethics committee (protocol n. BS/231009).

### Treatment strategy and follow up

Surgery was performed within one month after MR, unless medical complications, preoperative malnutrition and/or associated comorbidities required more prolonged medical treatments. A detailed, step-by-step description of CTS has already been published by our group [6]. Contralateral neck dissection was performed in case of multiple clinical positive nodes and/or tumors approaching the midline. Adjuvant treatments were discussed on a case-by-case basis within our Institutional Multidisciplinary Tumor Board. In general, adjuvant radiotherapy was indicated in case of close margins (< 5 mm), pT3 or higher, and 2 or more positive lymph nodes. Concurrent chemotherapy was administered in case of extranodal extension and/or positive margins.

Follow-up included clinical examination every 2 months during the first year, every 4 months during the second, and every 6 months thereafter. Head and neck imaging was performed every 6 months for 3 years and then annually up to the fifth year after treatment. Chest computed tomography or positron emission tomography were performed annually for 5 years.

### Statistical analysis

Variables included in the analysis were expressed in terms of median, interquartile range (IQR), range of values, and percentages. Since continuous variables showed a non-normal distribution, non-parametric Wilcoxon-signed rank test and Spearman’s correlation coefficient were used, as appropriate. Agreement between radiological and pathological measures was graphically represented using the Bland-Altman plot.

Outcomes of interest were OS, defined as the time from surgery to death from any cause or latest available follow-up (censored observations), and LRRFS, defined as the time from surgery to the first local or regional relapse whichever occurred first or latest available follow-up (censored observations).

For continuous variables, linear assumption was first assessed through plot inspection of Martingale residuals. A linear relationship between a continuous variable and the outcome of interest was further tested through restricted cubic splines (RCS) models, testing the non-linear component by Chunk tests and plots inspection. All continuous variables tested showed a linear relationship when plotted against 2-year probability of OS and LRRFS.

pDOI and pTDFM were categorized according to the following widely accepted cut-offs:

- pDOI > 10 mm (at least pT3) vs. pDOI ≤ 10 mm (4),
- pTDFM ≥ 5 mm (R0 free margins) vs. pTDFM > 0 and < 5 mm (R0 close margins) [10].

Survival estimates with relative 95% confidence interval (CI) for the entire cohort of patients and according to the most relevant variables were obtained and compared through the log-rank test. Survival curves were plotted using the Kaplan-Meier method. The clinical, radiological, and pathological variables of interest were tested using the Schoenfeld residuals to check the proportional assumption and further analyzed through uni- and multivariable Cox proportional hazards model; results were expressed in terms of hazard ratio (HR) and 95% CI.

Statistical analysis was performed using R (version 4.2.1, R Foundation for Statistical Computing, Vienna, Austria); p values < 0.05 (two-tailed) were considered statistically significant.

## Results

Sixty-four patients met the selection criteria and were included in the present study. Median age was 61 years (IQR, 45-73.2); 68.7% of patients were males. A slight prevalence of right OTSCC was observed (59.4%). Surgery followed preoperative MR after a median of 19.5 days (IQR, 10.7–30.2). Further details on descriptive statistics are available in Table 1.

**Table 1:** Descriptive statistics

Variable	% (*n*)
Gender	
Male	68.7 (44)
Female	32.3 (20)
Clinical stage	
II	15.6 (10)
III	59.4 (38)
IVA	23.4 (15)
IVB	1.6 (1)
Side	
Left	40.6 (26)
Right	59.4 (38)
Clinical T category	
cT2	21.9 (14)
cT3	78.1 (50)
Clinical N category	
cN0	59.4 (38)
cN1	15.6 (10)
cN2	23.4 (15)
cN3	1.6 (1)
Neck dissection	
Bilateral neck dissection	25 (16)
Unilateral neck dissection	100 (64)
Margins	
R0	71.8 (46)
R0 close	28.2 (18)
LVI +	39 (25)
PNI +	73.4 (47)
Extrinsic muscles involvement	75 (48)
TN tract involvement	20.3 (13)
Grading	
G1	12.5 (8)
G2	53.1 (34)
G3	34.4 (22)
Pathological T category	
pT1	1.6 (1)
pT2	26.6 (17)
pT3	71.8 (46)
Pathological N category	
pN0	48.4 (31)
pN1	20.4 (13)
pN2	15.6 (10)
pN3	15.6 (10)
Extranodal extension	
ENE+	17.2 (11)
Pathological stage	
I	1.6 (1)
II	12.5 (8)
III	56.2 (36)
IVA	15.6 (10)
IVB	14.1 (9)
Adjuvant therapy	
RT	43.7 (28)
CHRT	25 (16)
No adjuvant treatment	32.3 (20)
Local and regional recurrence	
T	7.8 (5)
N	6.3 (4)
T and N	7.8 (5)

*Legend*: R0, negative surgical margins (5 or more mm); R0 close, close surgical margins (less than 5 mm); LVI, lympho-vascular invasion; PNI, perineural invasion; T-N tract, tract including lymphatic vessels travelling from the tumor to the draining neck lymph nodes; ENE, extra-nodal extension; RT, radiotherapy; CHRT, chemoradiotherapy

### Radiological and pathological analysis

**Fig. 2:**
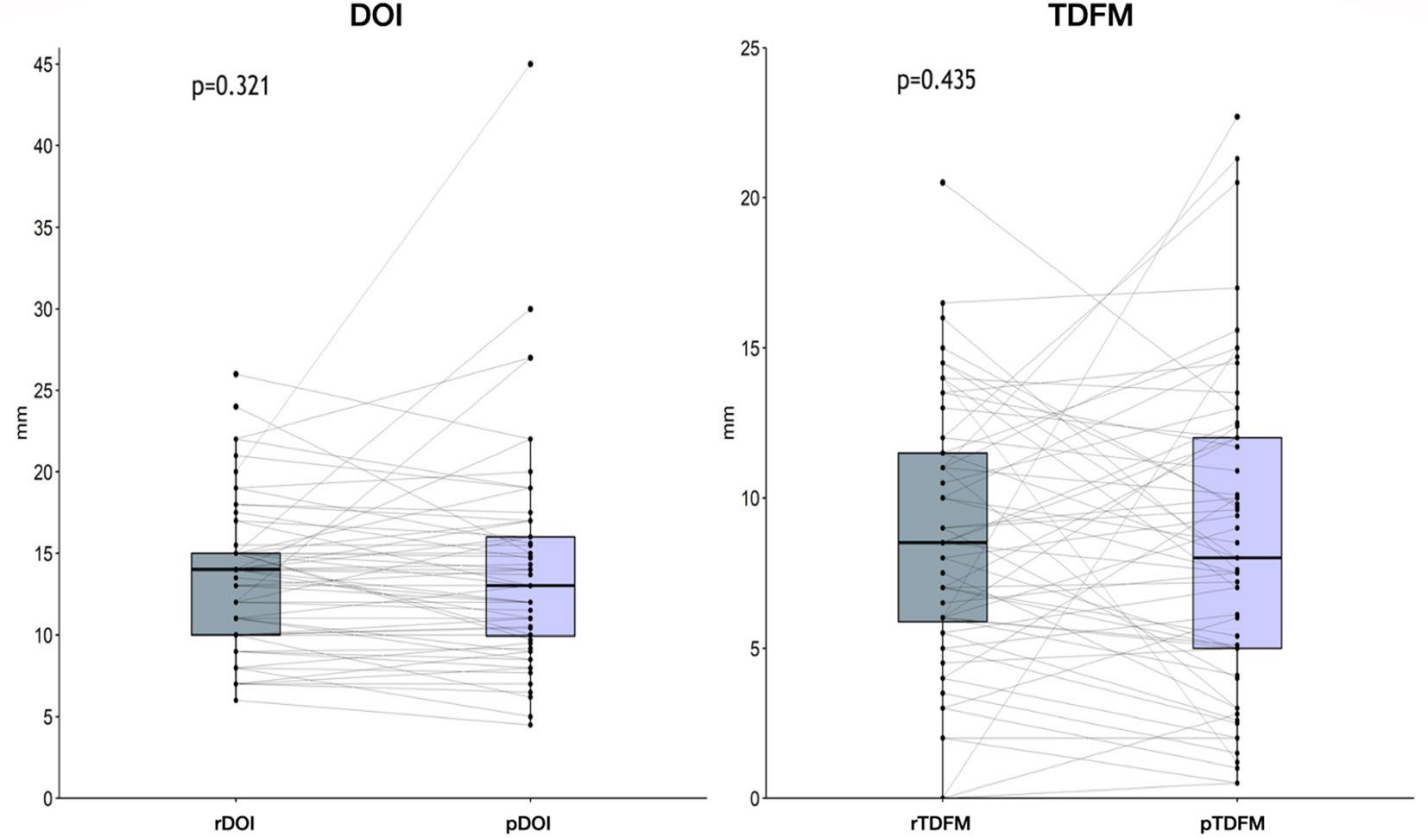
Box and whisker plots comparing the radiological and pathological values of depth of invasion (DOI) and tumor distance from midline (TDFM)

**Fig. 3:**
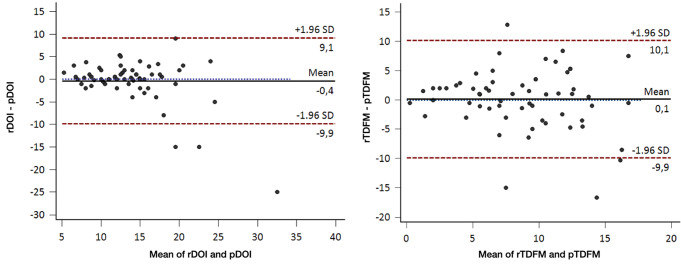
Bland-Altman plots showing the agreement between radiological and pathological depth of invasion (pDOI and r DOI) and tumor distance form midline (pTDFM and rTDFM)

Preoperative MR analysis showed a median rDOI of 14 mm (IQR, 10–15) and rTDFM of 8.5 mm (IQR, 5.8–11.5); review of histological slides returned a median pDOI of 13 mm (IQR, 9.9–16) and pTDFM of 8 mm (IQR, 5–12). No significant difference was observed between rDOI and pDOI (*p* = 0.321) or between rTDFM and pTDFM (*p* = 0.435) (Figs. 2 and 3).

Median DOId (rDOI-pDOI) was + 0.25 mm (IQR, -1 to + 1.62), whereas median TDFMd (rTDFM-pTDFM) was + 0.9 mm (IQR, -2.5 to + 2.5). No significant association was found between time elapsed from MR and surgery and DOId (*R* = 0.025, *p* = 0.850) and TDFMd (*R* = 0.210, *p* = 0.130).

### Survival analysis

The median follow-up was 42 months (IQR, 11.5–76). At the time of the end of the study, 27% of patients died, with a median survival of 28 months (IQR, 11–55), whereas 21.9% experienced at least one local or regional relapse, with a median disease-free interval of 9.5 months (IQR, 7.7–42.5). Eight (12.5%) local recurrences were observed; of these, 2 (25%) extended from the midline to the contralateral tongue compartment.

Two- and 5-year OS were 85.7% (95% CI, 77.9–95.4%) and 70.4% (95% CI, 58.1–85.4%), respectively; 2- and 5-year LRRFS were 84.3% (95% CI, 75.3–94.3%) and 76.1% (95% CI, 64.7–89.5%), respectively.

**Fig. 4:**
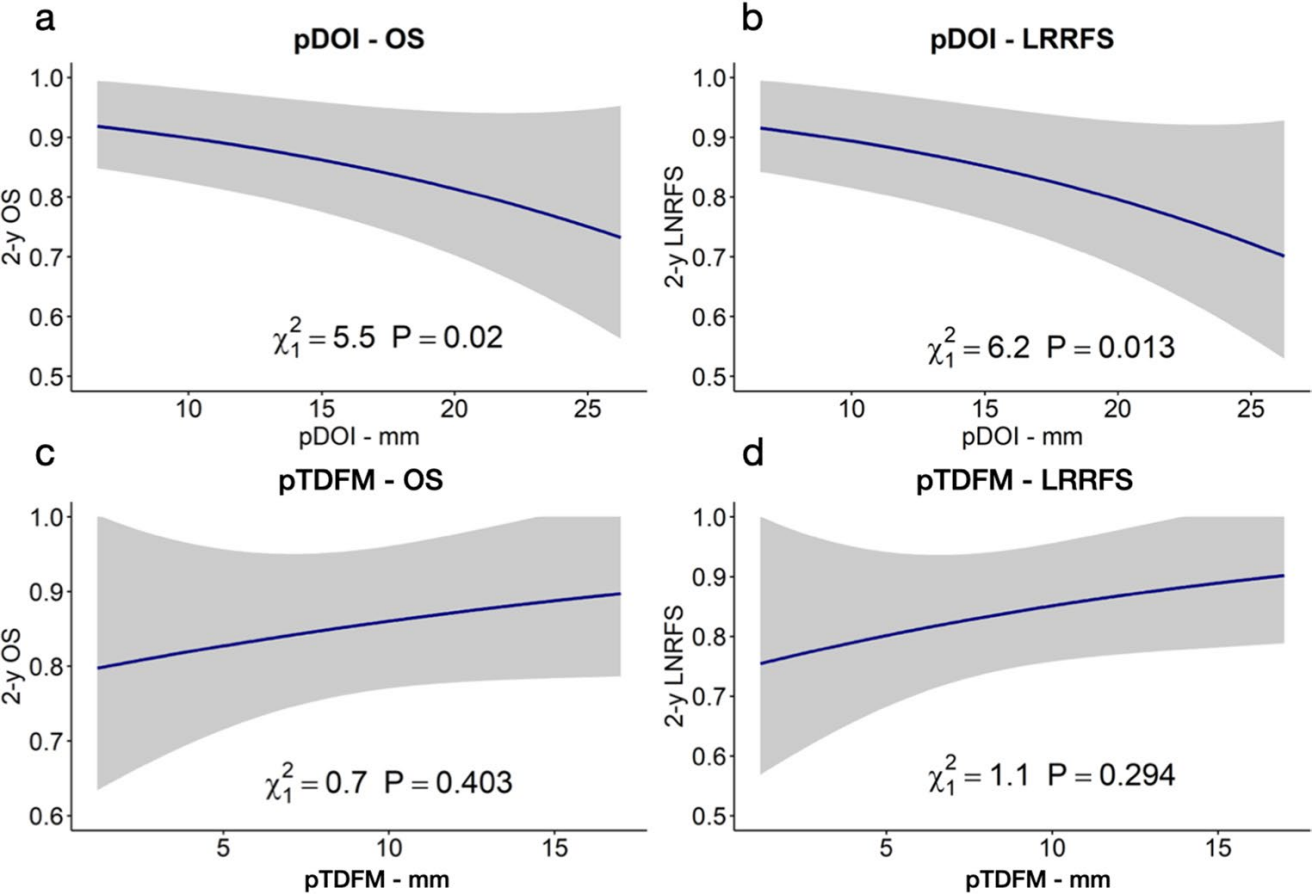
Linear models showing the significant correlation between pathological depth of invasion (pDOI) and 2-year overall survival (2-y OS) (Fig. 4a) and local and regional recurrence free survival (2-y LRRFS) (Fig. 4b), and the non-significant correlation between pathological tumor distance from midline (pTDFM) and 2-year OS (Fig. 4c) and LRRFS (Fig. 4d)

**Fig. 5:**
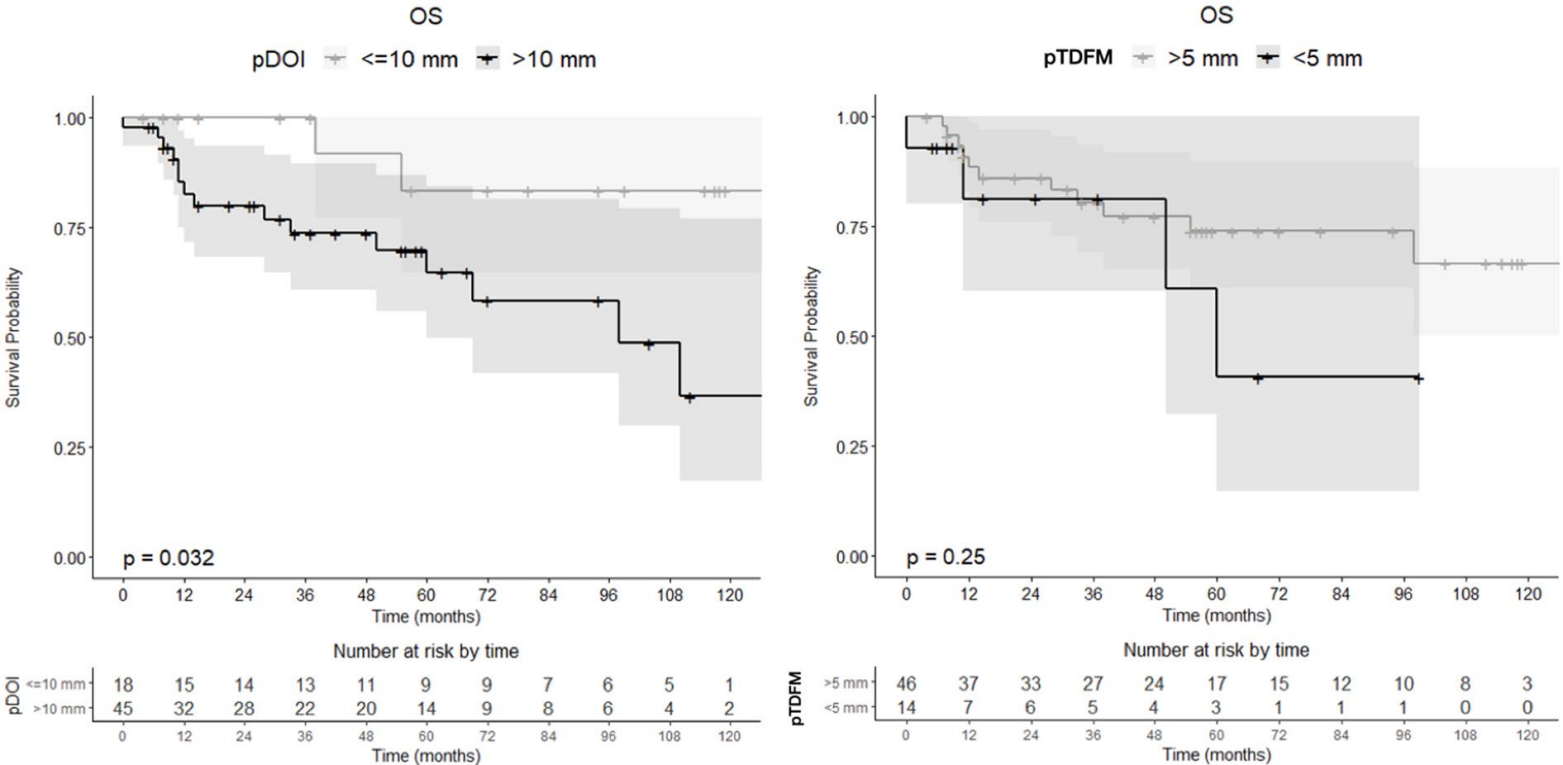
Kaplan Meier overall survival (OS) curve according to pathological depth of invasion (pDOI) and pathological tumor distance from midline (pTDFM)

A linear and significant relationship with 2-year OS (*p* = 0.020) and LRRFS (*p* = 0.013) was found for pDOI; although linear, the relationship between pTDFM was not statistically significant in terms of both 2-year OS or LRRFS (Fig. 4). Once categorized, pDOI > 10 mm was significantly associated with poorer OS (HR = 4–42, 95% CI 1-19.55) (Fig. 5), but not significantly associated with lower loco-regional control (*p* = 0.314). pTDFM < 5 mm (defined as R0 close margins for standard surgery) was not significantly associated with worse OS (*p* = 0.455) or LRRFS (*p* = 0.168) (Fig. 5).

Multivariable analysis (Table 2) confirmed that pDOI > 10 mm, but not pTDFM < 5 mm, was an independent prognosticator of OS, after adjustment for pN status and adjuvant treatment(s).

**Table 2:** Multivariable analysis

Overall survival			
Variable		HR (95% CI)	*p* value
**pDOI**	≤ 10 mm	Reference	0.048
	> 10 mm	7.82 (1.01–60.58)	
**pTDFM**	≥ 5 mm	Reference	0.100
	< 5 mm	3.07 (0.81-11-65)	
**Nodal status**	pN0	Reference	0.006
	pN+	8.13 (1.82–36.28)	
**Adjuvant treatment(s)**	None	Reference	
	RT	0.34 (0.09–1.32)	0.120
	CHRT	0.13 (0.02–0.85)	0.033

*Legend*: pDOI, pathological depth of invasion; pTDFM, pathological tumor distance from midline; RT, radiotherapy; CHRT, chemoradiotherapy; HR, hazard ratio; CI, confidence interval

## Discussion

The philosophy of CTS is to remove a tumor that is still confined in one hemitongue following precise and standard anatomical pathways known to negatively affect oncological outcomes. These pathways are represented by the extrinsic tongue muscles, lingual and hypoglossal nerves, blood and lymphatic vessels, as well as the areolar tissue forming the T-N tract at the level of the floor of mouth, potentially involved by tumor growth and spread [7]. In fact, CTS is aimed at removing the affected hemitongue compartment “en bloc” with the T-N tract and neck lymph nodes, by splitting the tongue along its midline raphe in order to avoid injury to the contralateral healthy hemitongue extrinsic muscles. Once provided that OTSCC is confined within one hemitongue compartment, the resection is guided by precise anatomical rules regardless of how the tumor extends into the hemitongue. Therefore, the distance between the tumor and resection margins can be extremely different along different directions.

The midline lingual raphe is defined as an avascular fibro-fatty tissue separating the extrinsic muscles of one compartment from the opposite one and is considered a fundamental landmark in CTS given that it is hypothesized to act as a barrier to contralateral tumor progression. In fact, midline involvement by advanced OTSCC, often associated with perineural invasion (PNI), is highly predictive of contralateral node metastases that independently affect both OS and disease-free survival (DFS) [11, 12]. Lloyd et al., in 5430 patients affected by OTSCC, noted that lateralized T1-T3 lesions had contralateral nodal metastases in 0.7–0.9% of cases, whereas T4 and T2-T3 tumors approaching the midline had corresponding rates of 8.3–13%. The authors concluded that primary extension across the midline was associated with a mean survival reduction of about half in lateralized OTSCC and this was also confirmed in multivariable analysis, thus highlighting that midline involvement is a strong predictor of decreased survival. In another recent publication by Akamatsu et al. [11] on 32 patients affected by cT3-4a OTSCC, in multivariate analysis midline involvement and PNI were independent predictors of contralateral nodal metastases, decreasing both OS and DFS.

In 1969, Feind and Cole [13] described three possible routes of OTSCC diffusion to the contralateral tongue compartment and neck: (1) dissemination from the primary lesion through pre-existing lymphatic vessels; (2) tumor spread beyond the midline from ipsilateral involved nodes *via* efferent collateral lymphatic vessels in case of high nodal burden; (3) primary tumor arising in or invading a central area of the tongue without a real midline barrier (e.g. at the level of the tip of this organ). In contrast to the above mentioned studies, Kurita et al., analyzing 129 patients affected by lateral OTSCC, did not find midline involvement to be an independent predictor of survival, but identified T category as one of the most important prognosticators [14].

One of the main criticisms of CTS has been classically represented by management of its medial (deep) surgical margin when the tumor approaches the midline lingual raphe and how this can effectively act as a protective barrier against contralateral neoplastic extension. In fact, in case of OTSCC with DOI ≥ 10 mm, the TDFM is frequently < 5 mm and this can result in a final histopathological rendering of “close margin”. The present study confirmed that pDOI is the major prognosticator in our cohort of patients, negatively affecting both OS and LRRFS, and confirming the utility of its introduction in the 8TNM for staging of OTSCC [4]. Interestingly, pTDFM does not seem to affect either OS or loco-regional control. Whereas the concepts of DOI and TDFM can be specular, the former represents a greater tumor aggressiveness in terms of volume and advancement towards neighboring structures especially within the paramedian septum [15].

Although still controversial, some authors consider the median lingual raphe as an anatomical barrier, where a < 5 mm margin may be equally considered oncologically safe compared to other areas. Even though the present work is underpowered to confirm this concept, TDFM proved to be a less relevant prognostic factor than DOI, thus supporting CTS at least in patients without tumors reaching the midline. Of note, in a recent publication by Zanoni et al. retrospectively evaluating 381 tumor specimens of OTSCC treated by primary surgery, the authors found that the optimal surgical margins cut-off associated with LRFS was 2.2 mm. Patients with margins of 2.3 to 5 mm had similar LRFS to patients with margins greater than 5 mm (HR, 1.31; 95% CI, 0.58–2.96), and all other comparisons were significantly different (HR for positive margins, 9.03; 95% CI, 3.45–23.67; HR for 0.01 to 2.2 mm margins, 2.83; 95% CI, 1.32–6.07). Their conclusion was that 2.2 mm might be used as a new definition of close margins, stratifying the risk for local recurrence better than the arbitrary 5 mm cut-off that is presently used [18]. These results were recently confirmed by Solomon et al. on 187 oral cancer patients who were treated surgically, studying the impact of close margins on recurrence free and disease specific survivals. Their conclusion was that close margins do not affect oncologic outcomes compared to the most conventional risk factors, thus again hypothesizing a re-definition of the standard 5 mm cut-off for close margins [19].

Our analysis found a median pTDFM of 8 mm (IQR, 5–12), classically considered a good surgical margin, even though CTS has been frequently performed also for tumors closely approaching (but not reaching) the midline [5, 20]. Our survival analysis confirms that CTS can be considered an oncologically safe procedure that can guarantee optimal control of disease for selected treatment-naïve patients affected by cT2-T3 lateral tongue cancer. Moreover, the absence of correlation between TDFM and loco-regional recurrence can be considered as an indirect proof of the oncological soundness of CTS.

In this series we observed 12.5% of local recurrences and, of these, only 25% developed through the midline into the contralateral tongue. Interestingly, primary TDFM of these recurrent cases varied from 5 to 21 mm and those occurring through the midline had a TDFM of 21 and 9.7 mm, suggesting that field cancerization and/or satellitosis can always occur in OTSCC, irrespectively of TDFM and/or DOI.

Finally, an interesting finding of our paper was the strong collimation of radiological and pathological data on tumor infiltration, confirming that MR is a crucial imaging tool in predicting the neoplasm’s behavior. In a recent metanalysis by Li et al. [21], MR-based measures showed a significant overestimation of DOI, especially using T2-weighted sequences. This could be due to the inability to differentiate tumor from peritumoral inflammation or because of a suboptimal plane of measurement. In our study, rDOI was usually measured in the coronal plane on contrast-enhanced T1-weighted fat-saturated 3D sequences, thus allowing a measurement perpendicular to the tongue border. Differentiation between tumor and inflammation was then feasible using multi-sequence approach.

The primary limitation of the current study is its retrospective design and, due to the small sample size and low number of events (recurrences and deaths), results of our multivariate analysis need to be interpreted with caution.

## Conclusion

In selected T2-T3 lateralized OTSCCs, CTS can be considered as a safe and standardized oncological procedure. MR shows an excellent prediction of pDOI and pTDFM. At final pathology, DOI was the most relevant prognostic factor affecting OS, whereas TDFM did not affect survival outcomes. In case of lateral OTSCC not extending to the contralateral hemitongue, the median lingual raphe should be considered an oncologically safe margin even if the TDFM is less than 5 mm.

## Abbreviations

TDFM: Tumor distance from midline
DOI: Depth of invasion
CTS: Compartmental tongue surgery
OTSCC: Oral tongue squamous cell carcinoma
OS: Overall survival
LRRFS: Loco-regional recurrence free survival
DOI pDOI: Pathological
DOI rDOI: Radiological
8TNM: 8th edition of the TNM staging system
TDFM pTDFM: Magnetic resonance MR, pathological
TDFM rTDFM: Radiological
DOId: DOI difference
TDFMd: TDFM difference
IQR: Interquartile range
RCS: Restricted cubic splines models
CI: Confidence interval
HR: Hazard ratio
PNI: Perineural invasion
DFS: Disease free survival
